# Experimental Analysis of Nano-Enhanced Phase-Change Material with Different Configurations of Heat Sinks

**DOI:** 10.3390/ma15228244

**Published:** 2022-11-20

**Authors:** Hamza Fayyaz, Abid Hussain, Imran Ali, Hanzla Shahid, Hafiz Muhammad Ali

**Affiliations:** 1 Department of Mechanical Engineering, University of Engineering and Technology, Taxila 47050, Pakistan; 2 Department of Mechanical, Mechatronics and Manufacturing Engineering, University of Engineering and Technology Lahore, Faisalabad Campus, Lahore 39161, Pakistan; 3 Mechanical Engineering Department, King Fahd University of Petroleum and Minerals, Dhahran 31261, Saudi Arabia; 4 Interdisciplinary Research Center for Renewable Energy and Power Systems (IRC-REPS), King Fahd University of Petroleum and Minerals, Dhahran 31261, Saudi Arabia

**Keywords:** phase-change material, nanoparticles, heat sink, electronic device, passive cooling

## Abstract

The demand for high-performance and compact electronic devices has been increasing day by day. Due to their compactness, excessive heat is generated, causing a decrease in efficiency and life. Thermal management of electronic components is crucial for maintaining excessive heat within the limit. This experimental research focuses on the combined effect of nano-enhanced phase-change material (NePCM) with different configurations of heat sinks for cooling electronic devices. Multi-walled carbon nanotubes (MWCNTs) are used as nanoparticles with concentrations of 3 wt% and 6 wt%, RT-42 as the phase-change material (PCM), and aluminum as the pin fin heat sink material. Different configurations of the heat sink, such as circular, square, and triangular pin fins, are used against the fixed volume fraction of the fins. It is found that the square configuration has the highest heat transfer with and without PCM. A maximum base temperature reduction of 24.01% was observed in square pin fins with RT-42 as PCM. At 6 wt% of NePCM, the maximum base temperature lessened by 25.83% in the case of a circular pin fin. It is concluded from the results that a circular pin fin with NePCM is effective for base temperature reduction, and all fin configurations with NePCM collectively reduce the heat sink base temperature.

## 1. Introduction

The advancement in electronic equipment is increasing with time, and they are becoming more advanced day by day due to their sizes and weights. This technology is moving towards both a micro and nano level. Due to the increasing demand for more features, their compactness and operating speed will generate a lot of heat. Due to decreasing size, micro- or nano-level components produce heat, which may cause a reduction in efficiency. It requires a cooling system that controls heat; thus, the proper functioning of electronic devices is possible. The excessive heat generated in operating devices must be dissipated in a short interval of time, which makes it safe and increases their life span. The management of this heat produced in electronic equipment must be removed before it damages parts and can be used for the long term.

We already know that pure PCMs are not good conductors of heat. To improve the conduction of PCMs and enhance their heat transfer properties, high thermal conductivity nanoparticle materials are added. The purpose is to see the effect on the temperature of PCM with the addition of nanofluids, combined with a heat sink. Nano-enhanced PCMs can reduce the temperature and be added to see the additional benefit.

R. Kothari et al. [1] studied the effect of nanoparticles/PCM with different configurations of heat sinks for thermal management. The heat sink material is aluminum, with three different configurations (no fin, one fin, and three fins) along different nanoparticle concentrations. Results show that small concentrations of nanoparticles of NePCM are very effective. If we increase the concentration of nanoparticles there would be a depletion in latent heat. The results concluded if we decrease the concentration of PCM then it would apply to thermal management systems.

D. Sahel et al. [2] numerically investigated the hydrothermal performance of heat sinks with hemispherical pin configurations. The results of the numerical investigation are validated with the heat sink with a cylindrical pin fin. Then, the numerical investigation of solid hemispherical fins was compared with the previous results. There are six different perorations with different numbers of holes for HTPF. The better result of the hydrothermal performance factor was 1.98 for perforation with six holes. It was concluded that with the help of the perforation technique, there was a reduction in the pressure drop of the heat sink.

H. Muhammad et al. [3] experimentally investigated the optimization technique for heat transfer in electronic circuits by using PCM and different arrangements of heat sinks. Finding the most effective pin fin PCM arrangement is the goal of this investigation. There are different configurations of heat sinks rectangular, triangular, and circular. Each arrangement is filled with six different PCMs. The result was considered at different power levels. The analysis showed that without introducing PCM, triangular pin fins had a better result than others, and the same results were seen again after introducing PCM. However, the best PCM is RT-54 for triangular pin fin configuration. While studying the enhancement ratios, it was seen that SP-31 has the highest ratio, and the triangular pin fin design works well with and without PCM.

M. Junaid et al. [4] experimentally investigated the PCM base pin fin geometries for thermal conductivity enhancement. The heat sink, which is composed of aluminum, compared the cross-sectional areas of the fins on a circular and square basis and included inline and staggered arrangements of fins. Results showed that the square-staggered pin fin has a more efficient heat sink without PCM, whereas with the addition of PCM, the inline geometries of both configurations have a better result. The highest enhancement ratio at power level 5 for SP-31 was considered.

M.H. Joneidi et al. [5] experimentally evaluated the melting process of the heat sink with various arrangements of plate fins. The heat sink is made up of copper material and RT-35 is employed as a phase-change substance. At power level 5 W, the trend of the melting process and the variation in the temperature distribution of heat sink with different fin configurations is analyzed. There are 36 thermocouples positioned at different points to find the temperature and the height of the fins is 15 mm, 25 mm, and 35 mm. The finding indicates that by adding a greater number of fins, the melting rate will be increased and lowered base plate temperature during a phase transition. The temperature distribution profile is also uniform when the number of fins is increased. During critical temperature, fin increment gives efficient heat control of the heat sink base plate.

A. Arshad et al. [6] experimentally studied the impact of fin thickness and volume fraction on phase-change material for an increase in reliability. There are four configurations; the fin height is the same 20 mm, and both the finned and un-finned configurations were studied. To find out the thermal performance, the volume fraction is also varied. The finding showed that the base temperature of the plate is reduced by phase-change material and improves the operational time. The result also indicated that the effectiveness of heat sink/PCM largely depends on the number of fins. The comparison between the 3 mm fin thickness showed a better result than those with 2 mm thickness. The PCM-filled heat sink with a 2 mm fin thickness can provide the best thermal performance.

A. Muhammad et al. [7] used a numerical method to improve the design of the pin fin heat sink. For cooling the components of electronic devices, the heat transfer of air using porous pin fins is investigated. The impact on the heat sink is explored for various fins with aligned and staggered arrangements. The concluded results from all the case studies, with a high Darcy’s number and decreasing pin fin configuration, give a better performance for all. The aligned configuration has a higher value of performance evaluation criterion than the staggered one.

Y. Huang et al. [8] experimentally studied the thermal performance of a finned heat sink with PCM. There is also a comparison between the finned metal foam heat sink and a finned heat sink. Porosity and pore density effect on the thermal performance of finned metal foam sinks is investigated. When the porosity decreases, then the operating temperature of the sink is also low, and the reliability period is short. To obtain the optimum melting performance of the sink, a porosity of 0.9% is found to be good. A sink operates at a low temperature and maintains dependability when pore density is increased.

G. Alfalah et al. [9] numerically investigated the pin fin heat sink thermal performance while making it cost-effective for concentrating photovoltaic systems. The cell area, efficiency, and heat sink material are studied. Three arrangements of the heat sink are studied known as a traditional sink, circular pin fin heat sink and last one is cylindrical pin fin heat sink. For all three designs, there are 49 total fins, and the height is 30 mm. The findings demonstrated that the circular configuration has maximum thermal performance without exceeding the cell temperature. Economically, aluminum circular pin fin is less costly than copper.

N. Putra et al. [10] experimentally investigated the performance of the passive cooling of electric vehicle batteries by using heat pipe and phase-change material. Due to the advancements in electric vehicles, their capacity and maintenance is the main concern. The study consists of a finned L-shaped heat pipe and PCM, which are arranged parallel, so the heat pipe absorbs one side heat of the battery, and phase-change material absorbs another side of the heat and dissipates into the surroundings. The following three thermal management techniques were discussed without heat pipe, with heat pipe, and with heat pipe coupled with phase-change material. The results concluded that heat pipes quickly release battery system heat into the environment. The battery’s surface temperature decreased by a maximum of 26.6 °C. The battery system’s thermal performance is further improved by the incorporation of phase-change material into the heat pipe. The comparison of the analysis showed that RT-44 HC had a higher surface temperature than beeswax.

A.N. Desai et al. [11] numerically investigated the fin efficiency for PCM in a thermal control module. This study aims to investigate the most efficient fin configuration that limits the critical temperature of the thermal module. Five important parameters and six different fin configurations are investigated and then validated with results. It is concluded from the results that the temperature of the plate falls with more fins and is most feasible when the number of fins is 100. By raising the fin count, heat diffusion in phase-change material enhances and the critical temperature value is low. Triangular fin geometry has a higher area enhancement ratio and provides low critical temperature for the same percentage of fins. Triangular fin geometry with 100 fins and 20% mass of fins is considered the best configuration.

Y. Hu et al. [12] examined how the phase-change material is used in a ventilated window for heating and cooling purposes. Two ventilated windows are used for experimentation in which one of which is a PCM-based heat exchanger that is used for heat sink pre-cooling applications. The material cooled down at night and the cold phase-change material cools down the ventilation, whereas the PCM stores thermal energy during the daytime and releases heat for ventilation at night. The comparison of average ventilation to normal ventilation is 0.7 MJ per day. It is concluded from the results that the phase-change heat exchanger increases the ventilated window inlet air temperature to 2 °C for 12 h. The self-cooling mode of ventilated windows decreases the surface temperature of the glass to 0.8 °C.

N. Joshy et al. [13] experimentally investigated the effect of vibration on the PCM-based closed-pack thermal management battery system. At different discharge rates, a series of experimental tests were performed to study the transient thermal behavior of the battery pack. The study considers a discharge range of 3C to 5C and an amplitude of 30 to 50 mm/s. It is concluded from the results that the discharge rate has a greater effect on the temperature of the battery. When the discharge rate is low, the frequency effect on battery temperature is significant.

S. Hekmat and G.R. Molaeimanesh [14] experimentally investigated the thermal management of Li-ion batteries by using phase-change material and water pipe. The heat transfer rate of the phase-change material is very low. To overcome it, a hybrid thermal management system is employed, namely, a combination of PCM and water-cooled pipes. The study consists of two hybrid thermal management systems with different arrangements. The active and passive thermal management system is compared to find out the effectiveness of hybrid systems. There is a total of seven cases discussed. During first the case, a maximum temperature of 58 °C is observed and when the phase-change material or silicon oil is introduced between the cells there is a decline in temperature to 45 °C and 32 °C. By constructing the hybrid thermal management system, a significant change in temperature reduction is observed due to the superiority of the hybrid thermal management system.

C.J. Ho et al. [15] studied the convective heat transfer on the heat sink employing NePCM. The heat sink consists of eight channels with a divergent angle of 1.38°. The NePCM is used as a working fluid. It is concluded from the results that due to the presence of NePCM there is pressure drop occurs and low Reynolds numbers enhance the heat transfer and reduce channel temperature. With a low Reynolds number, working fluid passing through a channel is very slow and nanoparticles absorb more heat; thus, the heat transfer enhancement is 82% compared with using simple water as a working fluid. A high concentration of particles is not advantageous when the Reynolds number is high because fluid passes through a heat channel very quickly and cannot absorb much heat.

C. Yadav and R.R. Sahoo [16] examined the thermal performance and thermophysical parameters of multiwall carbon nanotube using a T history method. The specific heat capacity and thermal conductivity of capric acid phase-change material is increased by adding a 0.02 percent volume fraction of multiwall carbon nanotubes. As a result, the thermal conductivity improvement in the solid and liquid phases is 31.2% and 14.4%, respectively. By using a dynamic light scattering method, particle size analyses for different volume fractions of multiwall carbon nanotubes. At 0.02% volume fraction, the specific heat capacity of solid and liquid multiwall carbon nanotube PCM is 52.8% and 35.91%, which surpasses the pure capric acid phase transition material.

C.J. Ho et al. [17] experimentally studied the thermal performance of a mini-channel heat sink by using hybrid aluminum oxide water nanofluid and phase-change material. The Reynolds number ranged from 133 to 1515 for different concentrations of additives. Hybrid suspension performance is largely dependent on the Reynolds number and critical velocity. The results concluded that hybrid suspension improves heat transfer rate in heat sink as compared to base fluid due to more thermal conductivity of nanoparticles. By changing the hybrid suspension’s Reynolds number and nanoparticle concentration relative to the base fluid, better outcomes can be obtained.

A. Kumar et al. [18] experimentally described the thermal performance of NePCM for cooling electronic equipment. The addition of nanofluid to the heat sink is a passive cooling technique that eliminates the active cooling technique. The material used for the heat sink in this study is aluminum, with copper oxide as a nanoparticle and paraffin wax as a PCM. This study consists of the heat sink with different configurations such as circular, rectangular, and square with different heat flux and nanoparticle concentrations. According to the findings, adding nanoparticles to a phase-change material raises its heat conductivity and viscosity but reduces the latent heat of fusion. The enhancement in thermal conductivity and viscosity is 150%, and 100%, respectively, and the reduction found in latent heat of fusion is 24.57%. The maximum reduction in temperature was observed for heat sink with the square pin fin filled with PCM/NePCM. The results show that the highest enhancement ratio is obtained for the heat sink square pin fin.

C. Xu et al. [19] experimentally investigated the thermal performance of a microchannel heat sink using nanofluids. Thermal conductivity is enhanced by the addition of carbon nanotubes into paraffin wax. Nanofluid is added to a heat sink that absorbs and stores energy to exchange heat and provide cooling. Carbon nanotubes enhance the melting process but decrease the melting point, whereas carbon nanotubes increase the thermal conductivity by 323% when the concentration is 15%. They also decrease the latent heat and specific heat, and the maximum enhancement in the Nusselt number is 34.9%. When the frequency is about 6 Hz, thermal resistance decreases by 19.0%, and the Nusselt number increases by 15.20%. Thermal performance is also affected by heating power and pump power; high heating power enhances the Nusselt number and slightly decreases thermal resistance, whereas high power has the opposite effect.

J. Wang et al. [20] This study consists of nanoparticles mixed with paraffin wax to prepare a nanofluid for the thermal management of a heat sink. Different concentrations of nanoparticles and heat supply were applied to investigate the thermal management of the heat sink. When aluminum oxide is added to paraffin, the melting ratio is increased. The results concluded that uniform temperature distribution is achieved for aluminum oxide composite phase-change material at 10 W power levels. Thus, the minimum thermal resistance is also achieved for 1 wt% of aluminum oxide composite PCM.

C.J. Ho et al. [21] examined the cooling performance of aluminum oxide/water nanofluids. The concentration of nanoparticles is 8 wt% for heat sinks in order to evaluate hydro-thermal characteristics of the mini-channel heat sink. The result showed that adding nanoparticles into pure water declines wall temperature and enhances the heat transfer effect. When we increase the Nusselt number, the Reynolds number will also improve. The maximum value of heat transfer effectiveness is 1.4. It is declared from the results that embedding MEPCM into the multi-channel heat sink is insignificant.

N.M.P. Estelle and P. Estelle [22] used carbon nanotubes to study the thermal performance of mini-channel heat sink due to their higher thermal conductivity. The study also investigated the effect of surfactants on the stability of nanofluids and their influence on thermal performance. The performance of a carbon nanotube nanofluid may be examined using various surfactants at various volume fractions. The results indicate that nanofluids with lignin as surfactants have better thermal performance than sodium polycarboxylate. This is concluded from the results that pressure drop increases by 28% to 29% for sodium polycarboxylate nanofluid with a volume fraction of 0.1%. Additionally, it is demonstrated that the circular cross-section heat sink performs better than the square cross-section heat sink when the volume fraction pressure drops rise.

V. Kumar and J. Sarkar [23] used water-based nanofluids to conduct an experimental study on the heat sink’s hydrothermal economic performance. Different nanoparticles are combined with water to prepare water-based nanofluids with a volume ratio of 50/50. Different phase-change materials are used to prepare nanofluids and then compare results with water-based nanofluids. Pressure drops increase with the addition of nanoparticles into the base fluid, and both density and viscosity increase. The results concluded that a good impact can be seen on the convective heat transfer coefficient and Nusselt number when we increase the flow rate and nanoparticle concentration. Thermal resistance will be decreased by increasing both flow rate and nanoparticle concentration. The coefficient of performance decreases with an increase in the Reynolds number. High stability can be attained by adding a small concentration of nanoparticles.

F. Rajaee et al. [24] showed how PCM and nanofluids were used experimentally to examine the thermoelectric generator. The dissipated heat from the photovoltaic panels can be harnessed by the thermoelectric generators. The hybrid system consists of thermal photovoltaic cells and thermoelectric generators. The phase-change material is used to cool the experimental setup, which improves the performance of the system. The experiment was conducted with different working fluids such as water, 0.25%, 0.5%, 1%, and 1% with the phase-change material. The results concluded that a hybrid unit with nanofluid increases by 10.9% of electrical power as compared to water. Both phase-change material and 1% of nanofluids enhanced the electrical efficiency by 4.5%. The exergy efficiency for phase-change material and alumina powder is the highest.

A. Alfaryjat et al. [25] showed how nanofluids are prepared with distilled water in which the concentration of nanoparticles is 0.5–2%. It is concluded from the results that with increasing concentrations and mass flow rate, there is a decrease in the base temperature of the heat sink. At a 2% concentration of nanoparticles, there will be a 4% and 6% decrease in the temperature of aluminum oxide and zirconium oxide.

M. Neyestani et al. [26] experimentally studied the thermal performance of heat sinks using nanofluids. Different concentrations of nanoparticles (0.1 wt% and 0.2 wt%) were dispersed in water by using two-step methods. The solution is sonicated for 3 h on an ultra-sonicator at 20 kHz. The findings indicate that the staggering geometry has a higher heat transfer rate than the in-line geometry. The comparison showed that a 35% reduction in wall temperature and 2.2-times larger Nusselt number in a porous heat sink. The thermal performance of the heat sink is strongly affected by changing the geometry of the heat sink.

G.S.S. et al. [27] detailed how deionized water is utilized as a base fluid in an experimental study of the hexagonal tube heat sink, and various nanoparticle concentrations are added as the supporting materials. The nanofluids are prepared by a two-step method and sonicated for 7 h. The temperature was monitored at the inlet and outlet pipes of nanofluids and base fluids by using j-type thermocouples. It was concluded from the results that by using nanoparticles in the base fluid, the effectiveness of the hexagonal heat sink was improved. Aluminum oxide with deionized water nanofluids shows the highest effectiveness and a high heat transfer coefficient. By increasing the volume flow rate, aluminum oxide nanofluids have a high Nusselt number compared with silicon dioxide and copper oxide.

Numerical investigation of hybrid nanocomposite phase-change material (HNCPCM) is carried out according to the findings of A. Arshad et al. [28]. Inside the RT-28HC, nanoparticles are employed to improve thermal performance. Different shape factor values and volume fraction ratios are used to find out the best results. The computational domain in this study uses a two-dimensional rectangular heat sink. ANSYS–FLUENT used to solve governing equations. Melting is improved by the addition of Ag-GO similarly thermal conductivity and effusivity values are increased. The shape factor of 16.1 and the ratio of 1:3 for Ag-GO particles shows the most effective results.

F. Najafi et al. [29] examined the thermal management of a printed circuit board by using NePCM and the fluid flow technique. Various parameters (heat flux, flow rate, PCM types, NePCM types) on properties of the heat sink are studied. Uniform heat flux of 4, 7, and 10 kW/m^2^ is generated by the PCB of size 8.5 × 7 cm. Thermal paste is used to reduce the thermal resistance between an aluminum heat sink and a copper plate. The results from this study showed that nanoparticles of TiO_2_ and Fe_3_O_4_, when added to the pure paraffin, enhanced the cooling ability of pure paraffin.

K. Dammak and A. El Hami [30] examined the pin fin heat sinks thermal optimization by using the kriging model. The PCM used in this work is paraffin wax. The methodology is a combination of finite element analysis in Ansys and kriging modeling in MATLAB. The main objective is to reduce the volume of the heat sink. Five mesh sizes were studied in this analysis to find accurate results. The heat sink is supposed to be insulated from all sides except the bottom. The result shows that the more the volume of the PCM, the more heat is absorbed by it. Compared with the baseline design of the heat sink, the design optimization reduced the maximum temperature by 30%.

N.S. Bondareva et al. [31] examined how nanofluids have proven themself in one of the best thermal conductive materials in electronic devices. The melt viscosity and thermal conductivity both rise because of the addition of nanoparticles. The results concluded that if the nanoparticles’ volume fraction is about 2%, then it is the most effective concentration because, at this stage, thermal conductivity and viscosity are increased. Moreover, if the nanoparticles’ volume fraction is increased, it causes negative effects on a system.

L. Zhao et al. [32] examined how two different phase-change materials are used to compare their thermal conductivity, and both have a low melting point. One of them is an alloy of Bi-Pb-Sn-Cd, and the other is organic stearic acid. Numerical integration was used to calculate the phase-change value of the mentioned PCMs. By electron microscopy, it was observed that Bi-Pb-Sn-Cd has good thermal conductivity with aluminum alloy and copper. Copper foam was also added to stearic acid to increase the thermal conductivity of stearic acid. Therefore, it is concluded that low-melting-point alloys can work more efficiently as a sink.

Carbon nanotubes, often termed as CNTs, possess excellent physiochemical properties, such as high thermal conductivity up to 6000 (W/mk). In recent years, CNTs have been inserted into poor thermal conductive materials such as PCMs, to enhance thermal conductivity and thermal storage capacity of resulting PCM composites. These PCM composites are employed for efficient thermal management of high-power automobile battery packs. PCM thermal conductivity enhancement through the insertion of nanotubes vary from 7% to 1000%. It can also be noted that adding 1.5 wt% of single-wall CNT and multi-wall CNT could increase thermal conductivity to 57% and 50%, respectively [33].

After analysing the recent research studies, it was deduced that PCM-based heat sinks are becoming more attractive as options in the thermal management of electronic devices. PCMs can absorb and release heat during melting and solidification phases and minimize overheating in electronics. Furthermore, PCM are known for their stable performance throughout the phase-change cycles. Therefore, this study aims to investigate and compare the effect of different concentrations of MWCNTs on the thermal performance of heat sink. In current research, we have used the two-step method for the preparation of nanofluids. Additionally, we studied the influence of three types of pin fin geometries on the thermal performance of PCM/NePCM-based heat sinks. This research was carried out in order to enhance the thermal efficiency and reliability of electronic devices. The conclusion was drawn as to which concentration of nanofluid is best with which pin fin configuration in terms of performance.

## 2. Experimental Setup

**Figure 1:**
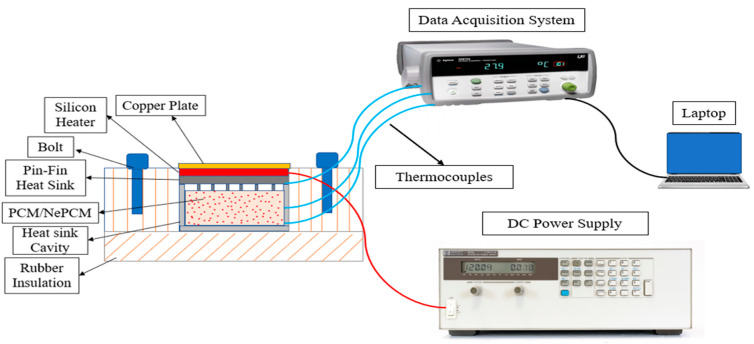
Schematic Diagram of Experimental Setup.

To examine the thermal management performance of PCM/NePCM-based pin fin heat sink, an accurate experimental setup was established. A schematic diagram of the original setup is shown in Figure 1.

In this research, the component that was under core consideration was the heat sink. Here, we used pin fin heat sinks of different configurations with the phase-change material and nano-enhanced phase-change material (multi-wall carbon nanotubes). Our focus was to give a more effective cooling technique for electronic devices. Here, we used the different configurations of heat sinks including square, circular, and triangular-shaped fins, and then with the addition of the phase-change material and nano-enhanced phase-change material. Using these different parameters, temperature variations were studied at different points inside the aluminum cavity. Our study took place in different phases. In the first phase, a different configuration of fins of heat sink was used. In second phase, pin fin configurations were used with phase-change material. In the third phase, pin fins were used with nano-enhanced phase-change material. Thus, during the experimentation, the temperature variations and the cooling process inside the cavity were observed using a data acquisition system and the thermocouple wires as the PCM and NePCM moves through the changes.

### 2.1. Pin Fin Heat Sinks

The heat sink material is aluminum; moreover, all three kinds of heat sinks are of the same material, i.e., aluminum’s geometry of circular fins, triangular fins, and square fins is designed in Solid Works, and then these shapes are processed in computer numerical control (CNC) to obtain the desired shapes.

For circular configuration, the total number of fins is 72. The base plate of the configurations for the circular fins has an area of (110 × 110) mm^2^, and the thickness of the base plate is 4 mm. Circular fins with a diameter of 4 mm are extruded up to 25 mm above the base plate. The centre-to-centre distance between circular pin fins is 11.6 mm from one side and 10.6 mm from the other. All fins are symmetrically designed and precisely distributed at equal distances from the corners. For square configuration, the total number of fins is 72. The base plate of the square fin’s configuration has an area of (110 × 110) mm^2^ and the thickness of the base plate is 4 mm. Square fins with a cross-sectional area of (4 × 4) mm^2^ are extruded up to 25 mm above the base plate. Centre-to-Centre distance between circular pin fins is 11.6 mm from one side and 10.6 mm from other side. The base plate of the triangular fin’s configuration has an area of (110 × 110) mm^2^ and the thickness of the base plate is 4 mm. All the fins are symmetrically designed and precisely distributed at equal distances from the corners.

**Figure 2:**
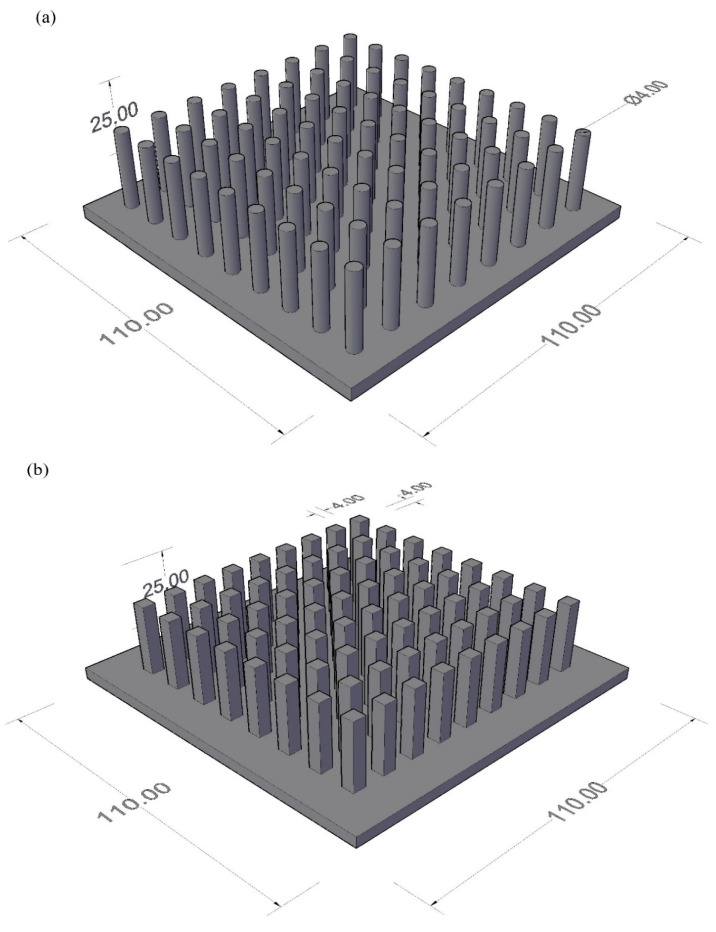

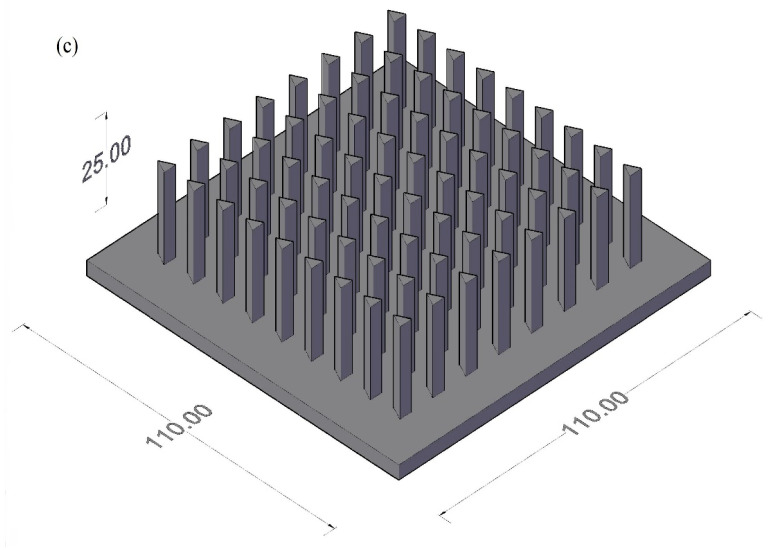
Heat sinks isometric views (**a**) circular, (**b**) square, and (**c**) triangular pin fins.

The material used for pin fin is Aluminum 2024. The configurations that we used for our experimentation were square, circular, and triangular types, as shown in Figure 2. The purpose of using these configurations was to find out the impact of shape on the results of thermal improvement. Heat sinks were used to absorb the heat from the electronic devices and transfer that heat to a cavity or the material present in the cavity.

The heat sink materials and dimensions is highlighted in Table 1 and Table 2 respectively.

### 2.2. Thermocouple Positioning

**Figure 3:**
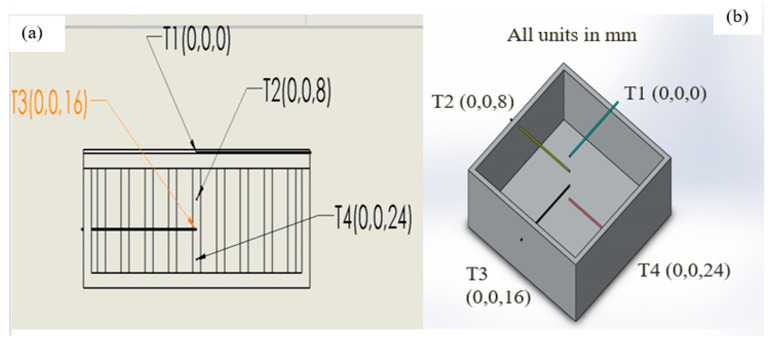
(**a**) Front view of thermocouples; (**b**) thermocouples coordinated in heat sink.

Highly sensitive and pre-calibrated 4 k type thermocouples are positioned from the top to the bottom of the heat sink to analyze heat dissipation. Thermocouples are fixed to heat sink side walls at different heights with temperature-resistant epoxy adhesive to make them immovable and leakproof. To measure the base temperature, thermocouple T1 is located between the heat sink and silicon pad heater. The thermocouple T2 is positioned at an 8 mm distance apart from the top of the heat sink, whereas T3 is positioned at 16 mm and T4 is positioned at 24 mm. For accurate temperature measurement within the heat sink, the thermocouples are implanted 55 mm inside the heat sink. The sides of the heat sink are insulated; thus, a unidirectional heat flow phenomenon is observed from the top to the bottom of the heat sink. The isometric view of the heat sink with the thermocouples’ location and coordinates of thermocouples inside the heat sink is shown in Figure 3 below.

### 2.3. Thermal Management Analysis

**Figure 4:**
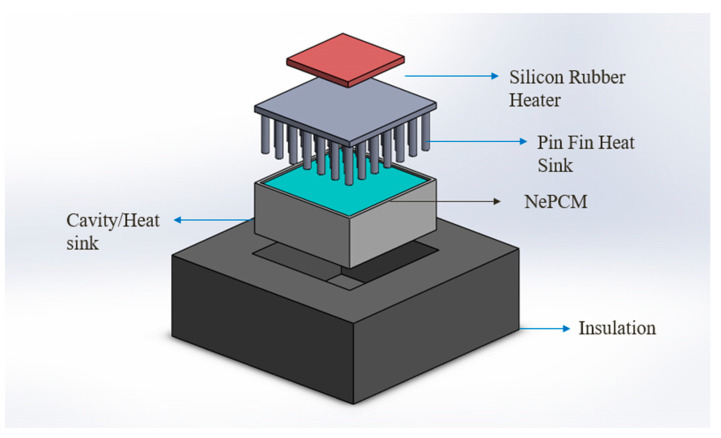
Exploded view of heat sink assembly.

A comparison between heating and cooling of the heat sink is conducted by employing different arrangements of the pin fin heat sink, e.g., (circular, square, and triangular) pin fins without PCM, (circular, square, and triangular) pin fins with PCM, pin fin configurations with NePCMs (3 wt%, 6 wt%). A power level of 10 W is used for this experimentation, and the heat sink is discharged in the same way it is charged, by insulating the sidewalls and base, enabling heat flow in only one direction, from top to bottom, for thermal analysis. The deconstructed view of heat sink assembly for the present study is shown in Figure 4.

### 2.4. Nanofluids

By dispersing nano-sized materials in base fluids, a new class of fluids known as nanofluids are produced. They are two-phase systems, with one phase (liquid phase) contained within another (solid phase). When compared with base fluids such as oil or water, nanofluids have been found to improve thermal characteristics such as thermal conductivity, thermal diffusivity, viscosity, and convective heat transfer coefficients. They have shown a wide range of prospective applications and approaches in a variety of disciplines. There are certain critical aspects to consider when designing a two-phase system. One of the most significant points is the stability of nanofluids and achieving the appropriate stability of nanofluids remains a great problem. Nanofluids have piqued the interest of scientists in recent years. The goal of this study is to focus on novel preparation methods and stability mechanisms, as well as new application trends for nanofluids and the heat transfer characteristics of nanofluids. Based on the study of these characteristics of nanofluids, we will try to identify some tough challenges that need to be resolved for future research.

### 2.5. NePCM Preparation

In this study, RT-42 PCM is used as a base fluid and Multi Walled Carbon Nanotubes (MWCNTs) are used as nanoparticles. The nano-enhanced phase-change material (NePCM) is prepared by using a two-step method. The concentrations of MWCNTs (Denton, TX, USA) explored in this study are 3 wt% and 6 wt% for PCM. The weight of the RT-42 PCM and nanoparticles are measured by digital analytical balance (Shimadzu ATY224, Kyoto, Japan).

During the synthesis of NePCM, firstly, PCM was melted by heating on a hot plate magnetic stirrer; the temperature is maintained at 90 °C, and then nanoparticles of various concentrations were inserted into the base molten PCM. RT-42 PCM/MWCNTs were continuously stirred at 900 rpm with a constant temperature of 90 °C on a hot plate magnetic stirrer (Corning PC-420D, New York, NY, USA) for 2 h, and then the Sodium dodecyl sulfate (SDS) surfactants (Sigma Aldrich, Waltham, MA, USA) along with one-third of MWCNTs were added into the mixture.

**Figure 5:**
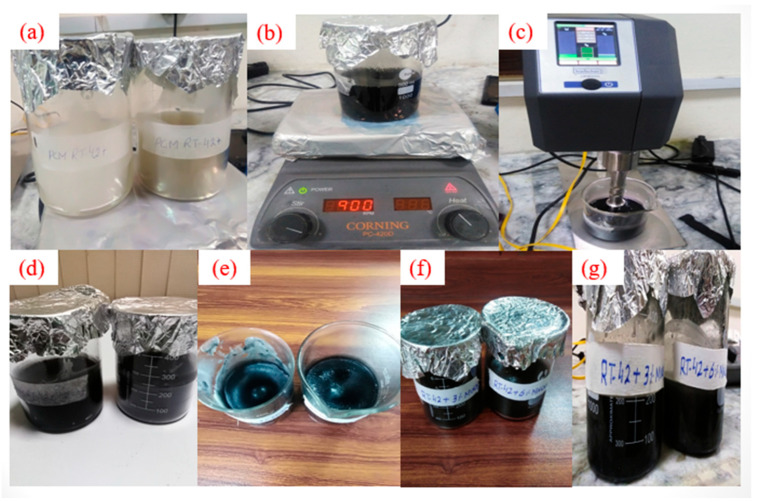
(**a**) RT-42 PCM heating, (**b**) magnetic stirring, (**c**) sonication, (**d**) prepared nanofluids, (**e**) NePCM after 24 h, (**f**) NePCM after 3 days, and (**g**) NePCM after 10 days.

Then, the mixture went through ultra-sonication using a Probe Sonicator (Hielscher UP400St, Teltow, Germany) at 25 KHz for 60 min. The ultrasonication improved the solubility of the nanoparticles in PCM, broke the particles into the mixture, removed agglomeration and dispersed the particles homogeneously. The synthesis procedure is shown in Figure 5 below. Finally, NePCM was poured into the heat sink and the stability of the prepared NePCM samples was analyzed visually. The samples of MWCNT NePCM with a concentration of 3 wt% and 6 wt% after preparation and after 24 h of preparation are displayed in Figure 5. 

## 3. Validation of Experimental Setup

**Figure 6:**
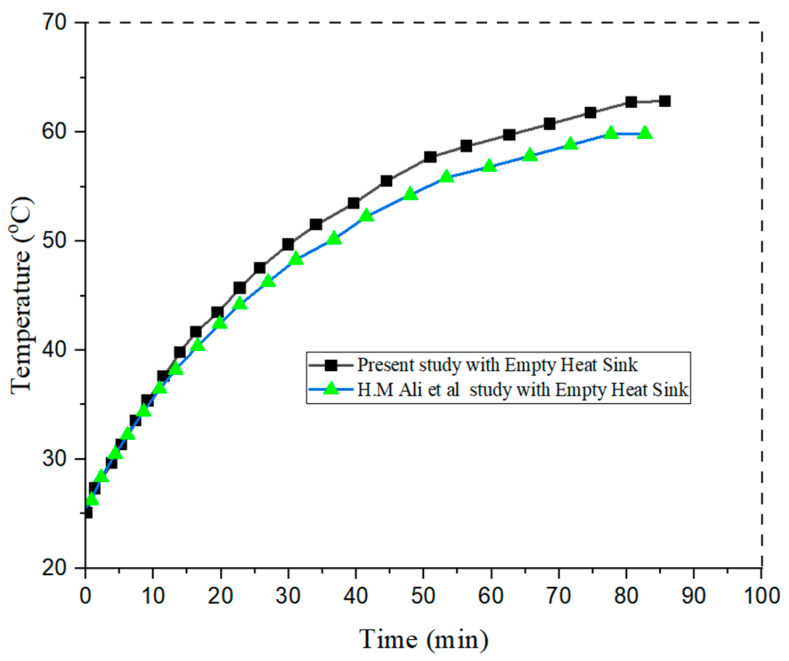
Validation with the experimental setup.

The experimental setup is verified by comparative analysis of an empty heat sink to those of previous studies conducted by H.M. Ali et al. [3], as shown in Figure 6. A heat sink with dimensions of 110 × 110 × 25 mm and a heating input of 10 W (0.82 kW/m^2^) is used to relate the results. It can be observed form the graph in Figure 6 that the variation in temperature with time shows the same trend as observed in previous studies. A deviation in temperature profile of 6% was observed for the study of empty heat sink. The small differences in results are due to different ambient temperature conditions, different materials, and heating input variations. 

## 4. Results and Discussion

### 4.1. Comparison of Different Configurations of Pin Fins (Square, Triangle, Circle)

**Figure 7:**
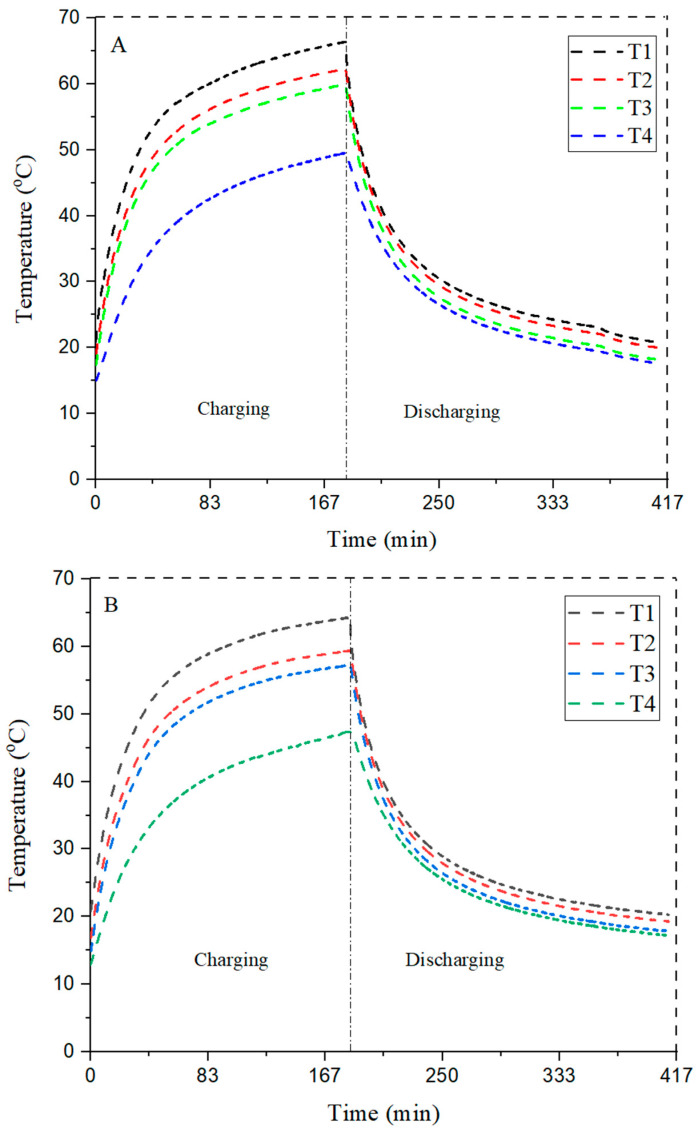

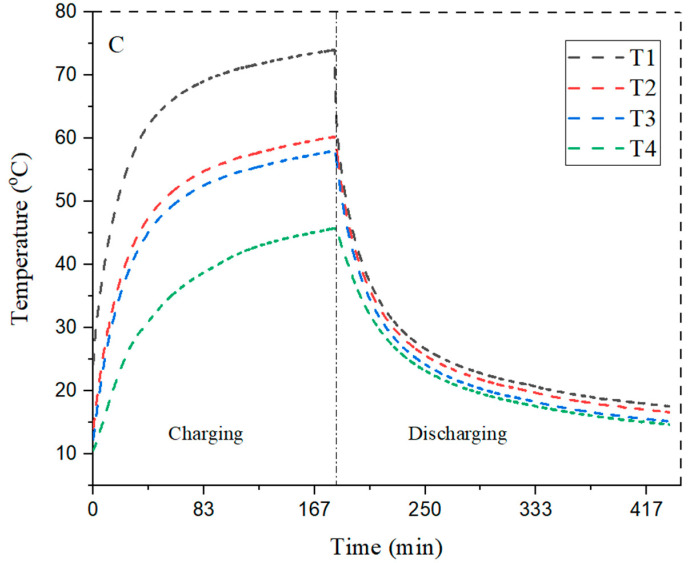
Comparison of different configurations without PCM (**A**) circular, (**B**) square, and (**C**) triangular fins.

The study consists of three phases: fins without PCM, fins with PCM, and fins with nano-enhanced phase-change material (NePCM). The fixed power level of 10 W is considered for all experiments. The first set of experiments is run for fins without PCM, and the comparison for fins is shown in Figure 7.

It can be observed from the graph that the three configurations of fins acquire a smooth curve and a clear constant difference shown between the plots during charging and discharging. Thermocouple T1 is located between the heater and heat sink at 0 mm, whereas T2 is located at 8 mm, T3 is located at 16 mm, and T4 is located at 24 mm; these distances are measured from the top of the heat sink, and this arrangement remained the same throughout the experiment. The graph was generated by the readings taken from these thermocouples. The maximum peak temperature of 63.3°C was observed at T2, which is close to the bottom of the heat sink where the maximum temperature reached 65.3 °C in the circular pin fin configuration. As T2 is very close to the bottom of the heat sink, a rapid change occurs and this change moves from layer to layer, from top to bottom as the heat is applied to the bottom of the heat sink; this is assuming that the heat travels in only one direction from the top to the bottom.

The results shown in Table 3 revealed that circular pin fin had less effective heat transfer characteristics as the maximum peak temperature is 63.3 °C, where the maximum temperature at the base of the heat sink is 65.3 °C. On the other hand, the square pin fin has better results relative to the circular pin fin, reaching a maximum peak temperature of 58.3 °C where the maximum temperature at the bottom of the heat sink is 64.3 °C. The difference between the line of both plots is quite narrow to each other. The triangular-shaped pin fin showed better results in heat transfer reaching a maximum peak temperature of 59.3 °C where the maximum temperature at the bottom of the heat sink is 73 °C.

Figure 7 shows the pattern of the charging and discharging phase of different configurations of pin fin heat sinks. The maximum temperature reduction is shown by T2 thermocouple considered here, because it is close to the base of the heat sink, showing a value of 58.3 °C. Furthermore, within the constant time span, the lowest temperature (18.287 °C) is also observed for the same. The square pattern is found to be the most suitable for temperature reduction in the absence of PCM.

### 4.2. Comparison of Different Configurations with PCM

PCM is introduced into the heat sink and studied the heat transfer effect on different configurations. The thermocouple positioning is the same, and 10 W heat is supplied by the heater. RT-42 is used as a PCM whose melting range is between 38–42 °C. The result shows a smooth graphical trend for all three heat sink configurations.

The data present in Table 4 tell us that a maximum peak temperature of 47.2 °C is achieved at T2, where the maximum base temperature of the heat sink is 52.1 °C for the circular pin fin configuration. The circular configuration with PCM shows the least efficient heat transfer, and graph plots are narrowed. From thermocouple T1 to T2, there is only a 4.9 °C change in temperature difference. Apart from this, the square configuration has the same trend; however, with T2, the maximum peak temperature is 44.3 °C, which is 3 °C less than the circular pin fin configuration. Thus, the square configuration has more heat transfer than circular pin fin with a maximum temperature difference of 14 °C between T1 and T4.

The triangular pin fin configuration has the highest heat transfer with a maximum peak of 45.4 °C when the maximum base temperature of the heat sink is 61.6 °C. The maximum temperature difference between T1 and T4 is 24 °C, which is more than the circular and square pin fin configurations. It is clear from the results that square pin fins with PCM have a better temperature reduction than triangular fins, which are better than the circular pin fins.

**Figure 8:**
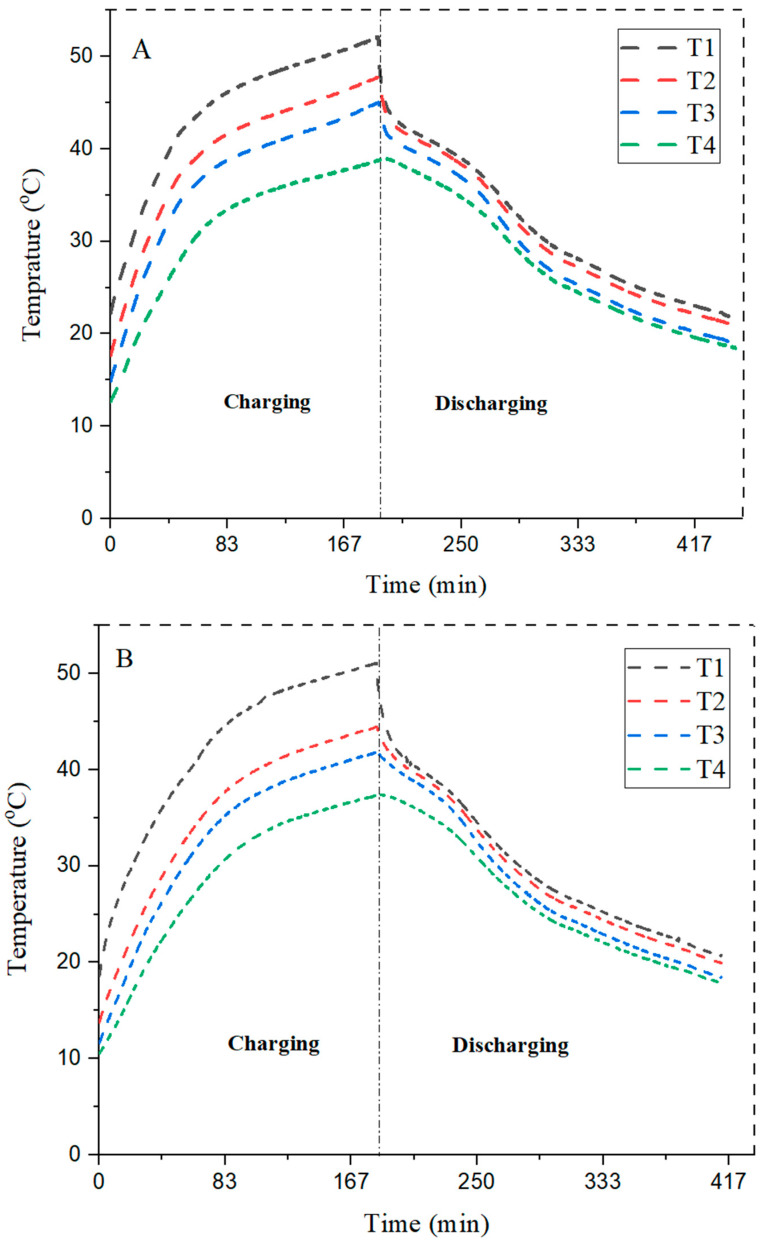

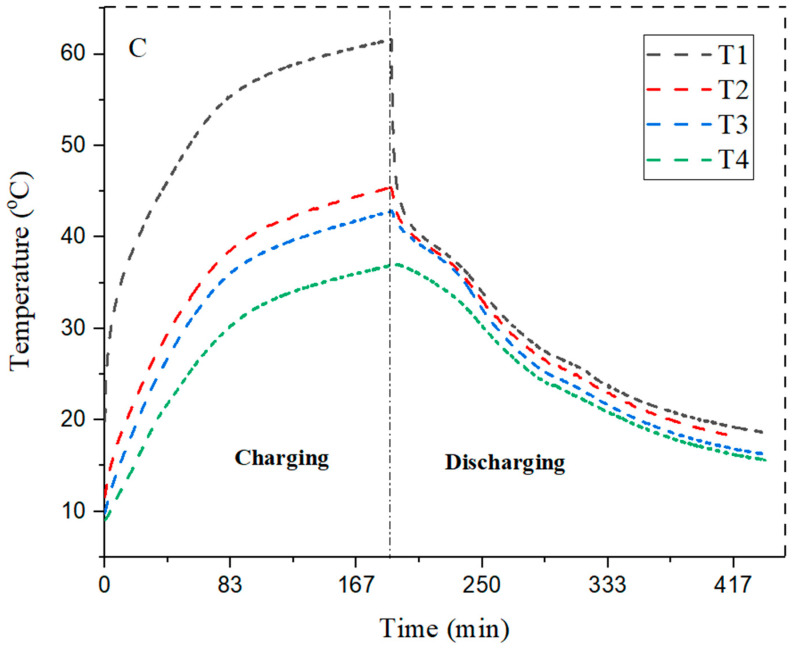
Comparison of different configurations with PCM (**A**) circular, (**B**) square, and (**C**) triangular fins.

Figure 8 delivers the results for different configurations of pin fin heat sinks for their charging and discharging phases in the presence of PCM to enhance the heat transfer rate. The results, among all, shows that the square fin module is the best, with a maximum peak temperature of 44.3 °C in the charging phase.

### 4.3. Comparison of Different Configuration with 3 wt%, and 6 wt% MWCNTs Concentrations

RT-42 PCM has very low thermal conductivity, MWCNT nanoparticles were used to increase the thermal conductivity. The preparation of the nano-enhanced phase-change material is carried out by the two-step method, which has already been discussed. Multi-walled carbon nanotube particles with weight compositions of 3 wt% and 6 wt% were added into the base fluid (RT-42 PCM).

With the addition of 3 wt% MWCNTs into the PCM, the heat transfer rate is improved compared with PCM, because we achieve a temperature of 44.5 °C at the bottom of heat sink T4,whereas in the case of RT-42 PCM it is 38.4 °C, as shown in Table 4 and Table 5. Square configuration with 3 wt% MWCNTs shows better results compared with a circular configuration with a heat transfer rate at T4 of 45.2 °C, whereas in the case of PCM it is 38.4 °C; however, triangular configuration with the same concentration shows less heat transfer than square configuration pointing T4 at 43.5 °C, whereas in the example without MWCNTs it is 37 °C.

**Figure 9:**
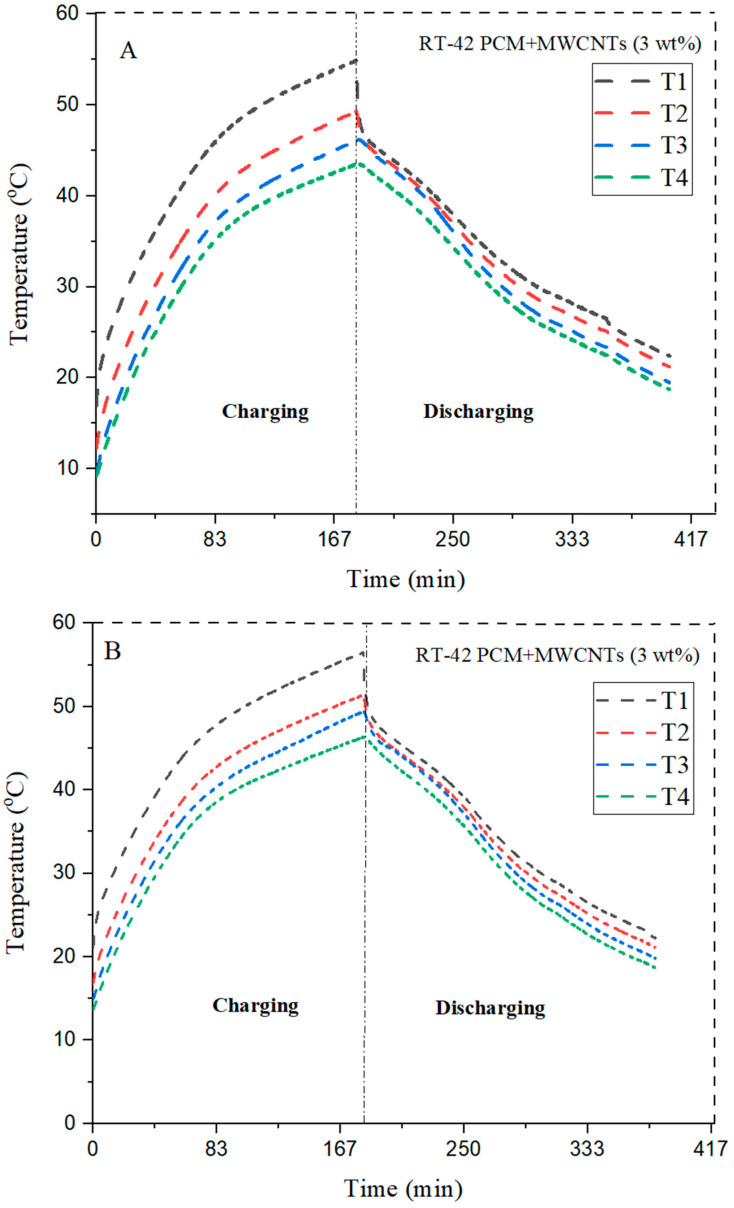

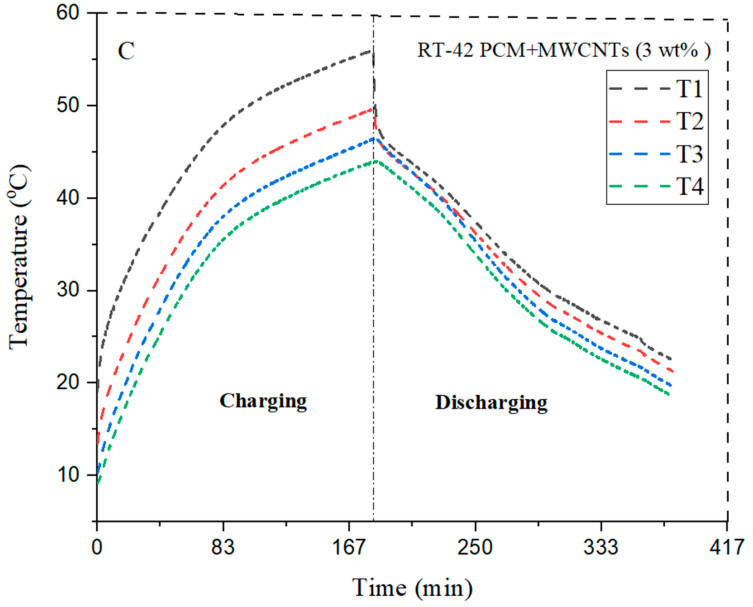
Comparison of different configurations with 3 wt% of NePCM (**A**) circular, (**B**) square, and (**C**) triangular fins.

Therefore, it can be concluded that 3 wt% MWCNT circular configurations showed more heat transfers than triangular fins, which is better than that of square pin fin NePCM. The maximum value at T4 is obtained in circular pin fins with 3 wt% NePCM at 44.5 °C, which means that a high heat transfer rate is observed during the same period of charging. The graph related to circular, square, and triangular fins with NePCM is shown below in Figure 9.

The effect of addition of 3 wt% MWCNTs is shown in Figure 9. The RT-42 PCM (paraffin wax) has very low thermal conductivity. To enhance the parameter, nanoparticles are embedded in the PCM. The results declare that circular configuration of pin fins is found to be the most suitable choice to enhance the cooling rate in the charging phase.

### 4.4. Comparison of Different Configurations with 6 wt% MWCNTs

Similarly, nanoparticles of 6 wt% MWCNTs were added into RT-42 PCM and showed effective heat transfer rates with different configurations.

From Table 6, it can be observed that heat transfer rate is improved by addition of 6 wt% MWCNTs. The circular pin fin configuration with 6 wt% NePCM has the least heat transfer rate in which at T4 maximum peak temperature is 39.4 °C whereas 3 wt% maximum peak temperature at T4 is 44.5 °C. Therefore, square pin fin with 6 wt% has improved the heat transfer rate with a maximum peak temperature at T4 of 39.3 °C, whereas the 3 wt% NePCM maximum peak temperature at the bottom layer T4 is 45.2 °C.

The triangular pin fin with 6 wt% of NePCM has showed small improvement in heat transfer with a maximum peak temperature at T4 of 39.2 °C, whereas the 3 wt% maximum temperature at the bottom layer is 43.5 °C. It is revealed from the results that the maximum temperature reduction is shown by the circular pin fin with 6 wt% of NePCM. With the addition of the concentration of nanoparticles, thermal conductivity is improved and enhances the cooling efficiency.

**Figure 10:**
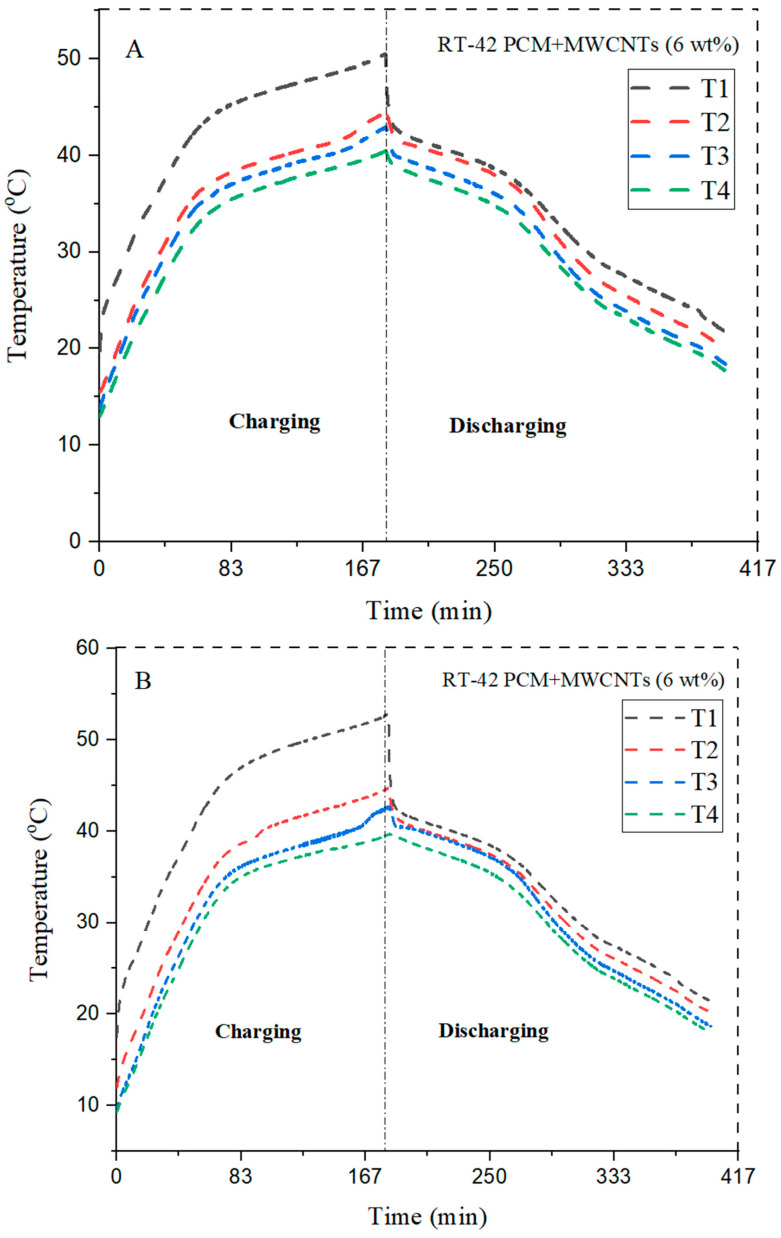

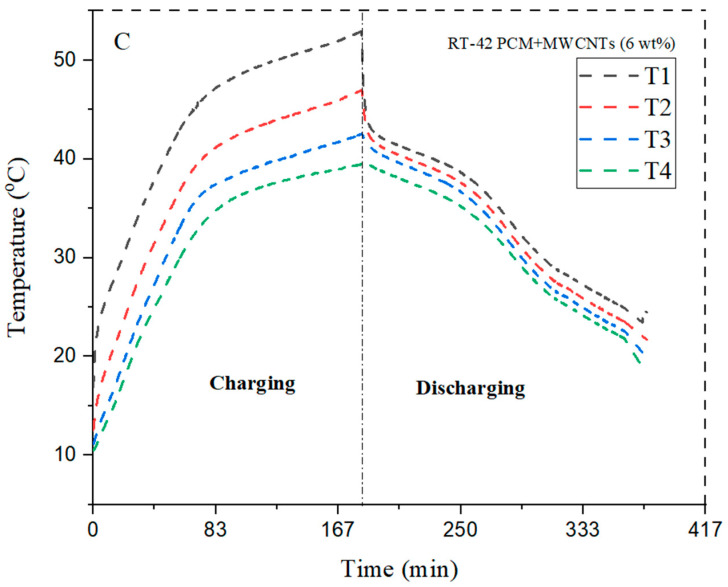
Comparison of different configurations with 6 wt% in a (**A**) circular, (**B**) square, and (**C**) triangular fins.

The quantity of nanoparticles has been increased and observed to see its impact on the enhancement of the thermal conductivity of PCM. Figure 10 shows the results of 6 wt% MWCNTs in the PCM. The trend shows that circular fins maximum reduces the base temperature and aid in efficient temperature reduction. The maximum temperature drop at the bottom of the heat sink is 43.9 °C in the case of circular fins, making it the best choice in the current scenario.

### 4.5. Comparison of Different Configurations (Circular, Square, Triangular) without PCM, with PCM and NePCM (3 wt%, 6 wt%)

The power level of 10 W, over a surface area of 110 × 110 mm^2^, described the heat flux of 0.8264 KW/m^2^ for three different configurations of heat sink (circular, square, triangular) pin fins; this, along with the weight percentage of nanoparticles (3 wt%, 6 wt%), has been chosen to find out the effect of NePCM on pin fin heat sinks and to find out the best configuration.

The figure below tells us the behavior of NePCM on different configurations of pin fin heat sinks at the same heat flux of 0.8264 KW/m^2^. It is revealed from the results that the maximum peak temperature of 63.29 °C is reached at the end of the charging process of the heat sink, without PCM and NePCM. Therefore, the heat sink shows uniform behavior during the charging and discharging process. The reduction in base peak temperature is seen by the addition of PCM and NePCMs. Among all, RT-42 based PCM with different configurations of the pin fin heat sink (circular, square, triangular) gave the best results and reduced the maximum base temperatures by 23.71%, 24.01%, and 23.38%, respectively. RT-42 PCM/MWCNTs with 3 wt% for (circular, square, triangular) pin fin heat sinks reduce the base temperature by 20.40%, 12.85%, and 16.97%, respectively. RT-42 PCM/MWCNTs with 6 wt% for (circular, square, triangular) pin fin heat sinks reduce the base temperature by 25.83%, 22.58%, and 21.60%, respectively.

It was concluded from the results that RT-42 PCM/MWCNTs with 6 wt% for circular pin fin configuration reduce the base temperature by up to 25.83% compared with square and triangular configurations with the same concentration. The best results are given by square pin fins with PCM reducing the base temperature by nearly 24.01%. Circular pin fins with 3 wt% of NePCM are given the maximum reduction of up to 20.40%, and the highest reduction in base temperature is shown in circular pin fins with 6 wt% NePCM are at 25.83%.

**Figure 11:**
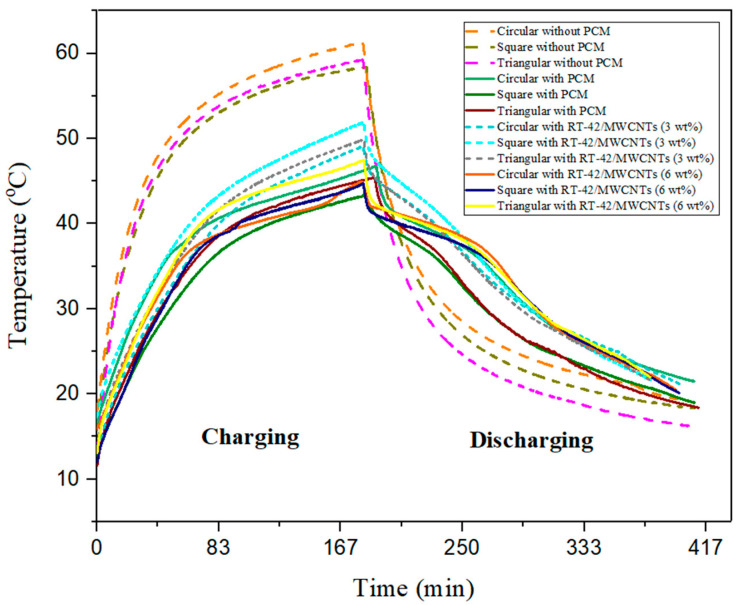
Comparison of different configurations for base temperature reduction without PCM, with PCM and NePCM (3 wt%, 6 wt%).

It was deduced from the above discussions that RT-42 PCM/MWCNTs with 6 wt% for circular pin fin configuration is the most efficient as the base temperature is reduced by up to 25.83%, whereas all the NePCMs collectively reduce the base temperature of the heat sink. Figure 11 shows the overall combined impact of heat sinks with different configurations of pin fins for thermal management. The impact of no PCM, PCM, PCM with 3 wt% MWCNTs and PCM with 6 wt% MWCNTs has been shown.

## 5. Conclusions

With the advancements in micro/nanotechnology, the electronic devices are becoming compact in size and shape along with the increasing demand for more features and fast operating speeds. Therefore, some efficient thermal management systems are required to control the generated heat and to ovoid overheating of the electronic devices. In this work, an experimental study was carried out to tackle this problem using heat sinks with different pin fin configurations saturated with PCM (RT-42) and MWCNTs with 3 wt% and 6 wt% concentrations. At a constant heat flux of 10W, the results shows that square configuration of the heat sink is a suitable choice at a base temperature reduction of 58.3 ℃ and 44.3 ℃ with and without PCM, respectively. At 3 wt% and 6 wt% of MWCNT in PCM, circular fins showed the best enhancement in the cooling rate with temperature reductions of 20.40% and 25.83%, respectively, among all options studied. Furthermore, this study indicates that the composite of nanoparticles and PCM also reduces the discharging time for the PCM and allows cooling to happen at a faster rate.

According to the author’s future recommendations, the thermal management of devices can be enhanced using nanoparticles; however, the main concern is to avoid agglomeration and to retain the structural stability of CNTs. Additionally, there is no specific criteria to define concentration that also need to be targeted. This can help to main the temperature of the heat sinks at the desired level, especially in the field of electronics.

## Figures and Tables

**Table 1:** Properties of heat sink material.

Thermal Conductivity K (W/m K)	120
Density p (g/cm^3^)	3
Specific Heat (KJ/kg K)	0.88

**Table 2:** Heat sink dimensions.

Dimensions	Circular Pin Fin	Square Pin Fin	Triangular Pin Fin
Base Plate Length (mm)	110	110	110
Base Plate Width (mm)	110	110	110
Center to Center Distance (mm)	6	6	6
Fin Height (mm)	25	25	25
Number of Fins	72	72	72

**Table 3:** Temperature data for configuration without PCM.

Configuration without PCM	Thermocouples	Starting Temperature (°C)	Maximum Peak Temperature (°C)	Ending Temperature (°C)
Circle	T1	20.719	65.393	20.884
	T2	19.055	63.292	19.043
	T3	17.145	58.727	16.932
	T4	15.521	49.024	15.844
Square	T1	20.184	64.335	20.267
	T2	16.796	58.384	17.287
	T3	15.016	57.805	18.311
	T4	13.903	47.271	18.125
Triangular	T1	24.126	73.045	19.871
	T2	14.038	59.281	16.558
	T3	12.776	56.685	14.655
	T4	10.547	45.788	13.621

**Table 4:** Temperature data for configuration with PCM.

Configuration with PCM	Thermocouples	Starting Temperature (°C)	Maximum Peak Temperature (°C)	Ending Temperature (°C)
Circle	T1	23.226	52.119	22.837
	T2	18.641	47.203	20.214
	T3	15.189	44.723	17.986
	T4	13.189	38.435	16.908
Square	T1	18.544	51.803	19.716
	T2	14.652	44.385	18.818
	T3	12.199	42.357	17.751
	T4	10.807	38.427	16.987
Triangular	T1	20.132	61.626	20.746
	T2	12.603	45.462	18.642
	T3	10.311	43.381	16.763
	T4	9.835	37.004	14.598

**Table 5:** Temperature data for configuration with 3 wt% MWCNTs.

Configuration with 3 wt% MWCNTs	Thermocouples	Starting Temperature (°C)	Maximum Peak Temperature (°C)	Ending Temperature (°C)
Circle	T1	17.391	54.911	21.406
	T2	13.743	48.791	20.394
	T3	10.243	46.692	18.764
	T4	9.868	44.578	17.685
Square	T1	20.245	56.135	21.264
	T2	16.305	51.905	20.132
	T3	15.851	48.424	18.855
	T4	13.674	45.223	16.743
Triangular	T1	18.111	55.114	22.653
	T2	14.933	49.121	20.781
	T3	10.628	46.987	19.227
	T4	9.806	43.506	17.792

**Table 6:** Temperature data for configuration with 6 wt% MWCNTs.

Configuration with 6 wt% MWCNTs	Thermocouples	Starting Temperature (°C)	Maximum Peak Temperature (°C)	Ending Temperature (°C)
Circle	T1	19.557	50.102	21.223
	T2	15.383	43.915	19.891
	T3	14.483	43.456	18.793
	T4	13.997	39.468	17.687
Square	T1	18.151	53.796	21.369
	T2	12.446	44.832	18.635
	T3	10.049	43.731	17.842
	T4	9.008	39.363	17.352
Triangular	T1	17.923	53.891	22.443
	T2	13.086	46.965	21.187
	T3	11.666	43.119	19.425
	T4	10.527	39.269	18.914

## Data Availability

Not applicable.

## Nomenclature

Main symbols	
G	Thermal Conductance (W/k)
HZ	Hertz (HZ)
I	Current (A)
K	Thermal Conductivity (W/m K)
P	Power (W)
q	Heating Input (kW/m^2^)
T1–T4	Thermocouple Location
T	Temperature (°C)
V	Voltage (V)
V_PCM_	Volume of PCM (mm^3^)
V_s_	Volume of Heat Sink (mm^3^)
Wt%	Weight Percentage
Abbreviations	
HS	Heat Sink
HNC	Hybrid Nanocomposite
MWCNTs	Multi Walled Carbon Nanotubes
NePCM	Nano-Enhanced Phase-Change Material
NPs	Nanoparticles
PCM	Phase-Change Material
RT	Rubitherm
TC	Thermal Conductivity
CNC	Computer Numerical Control
SDS	Sodium Dodecyl Sulfate

## References

[1] Kothari R., Sahu S.K., Kundalwal S.I. (2021). Chemical Engineering and Processing—Process Intensification Investigation on thermal characteristics of nano enhanced phase change material based finned and unfinned heat sinks for thermal management system. Chem. Eng. Process. Process Intensif..

[2] Sahel D., Bellahcene L., Yousfi A., Subasi A. (2021). Numerical investigation and optimization of a heat sink having hemispherical pin fins. Int. Commun. Heat Mass Transf..

[3] Ali H.M., Ashraf M.J., Giovannelli A., Irfan M., Irshad T.B., Hamid H.M., Hassan F., Arsha A. (2018). Thermal management of electronics: An experimental analysis of triangular, rectangular and circular pin-fin heat sinks for various PCMs. Int. J. Heat Mass Transf..

[4] Junaid M., Muhammad H., Usman H., Arshad A. (2017). Experimental passive electronics cooling: Parametric investigation of pin-fin geometries and efficient phase change materials. Int. J. Heat Mass Transf..

[5] Joneidi M.H., Rahimi M., Pakrouh R., Bahrampoury R. (2020). Experimental analysis of Transient melting process in a horizontal cavity with different configurations of fins. Renew. Energy.

[6] Arshad A., Ali H.M., Ali M., Manzoor S. (2017). Thermal performance of phase change material (PCM) based pin-finned heat sinks for electronics devices: Effect of pin thickness and PCM volume fraction. Appl. Therm. Eng..

[7] Mohammad A., Pouransari Z., Siavashi M. (2021). Improved design of heat sink including porous pin fins with different arrangements: A numerical turbulent flow and heat transfer study. Appl. Therm. Eng..

[8] Huang Y., Sun Q., Yao F., Zhang C. (2020). Experimental Study on the Thermal Performance of a Finned Metal Foam Heat Sink with Phase Change Material Experimental Study on the Thermal Performance of a Finned Metal Foam. Heat Transf. Eng..

[9] Alfalah G. (2020). Optimization and feasibility analysis of a microscale pin-fins heat sink of an ultrahigh concentrating photovoltaic system. Int. J. Energy Res..

[10] Putra N., Fahrizal A., Ariantara B., Abdullah N., Meurah T., Mahlia I. (2020). Case Studies in Thermal Engineering Performance of beeswax phase change material (PCM) and heat pipe as passive battery cooling system for electric vehicles. Case Stud. Therm. Eng..

[11] Desai A.N., Gunjal A., Singh V.K. (2020). Numerical investigations of fin efficacy for phase change material (PCM) based thermal control module. Int. J. Heat Mass Transf..

[12] Hu Y., Heiselberg P.K., Guo R. (2020). Energy & Buildings Ventilation cooling/heating performance of a PCM enhanced ventilated window—An experimental study. Energy Build..

[13] Joshy N., Hajiyan M., Siddique A.R.M., Tasnim S., Simha H., Mahmud S. (2020). Experimental investigation of the effect of vibration on phase change material (PCM) based battery thermal management system. J. Power Sources.

[14] Hekmat S., Molaeimanesh G.R. (2020). Hybrid thermal management of a Li-ion battery module with phase change material and cooling water pipes: An experimental investigation. Appl. Therm. Eng..

[15] Ho C.J., Liu Y., Yang T., Ghalambaz M., Yan W. (2021). Convective heat transfer of nano-encapsulated phase change material suspension in a divergent minichannel heatsink. Int. J. Heat Mass Transf..

[16] Yadav C., Sahoo R.R. (2020). Experimental analysis for optimum thermal performance and thermophysical parameters of MWCNT based capric acid PCM by using T-history method. Powder Technol..

[17] Ho C.J., Chen W., Yan W., Amani P. (2018). Contribution of hybrid Al_2_O_3_-water nanofluid and PCM suspension to augment thermal performance of coolant in a minichannel heat sink. Int. J. Heat Mass Transf..

[18] Kumar A., Kothari R., Sahu S.K., Kundalwal S.I. (2021). Thermal performance of heat sink using nano-enhanced phase change material (NePCM) for cooling of electronic components. Microelectron. Reliab..

[19] Xu C., Xu S., Daba R. (2021). Experimental investigation of thermal performance for pulsating flow in a microchannel heat sink filled with PCM (paraffin/CNT composite). Energy Convers. Manag..

[20] Wang J., Yu K., Duan R., Xie G., Sund B. (2021). Enhanced thermal management by introducing nanoparticle composite phase change materials for cooling multiple heat sources systems. Energy.

[21] Ho C.J., Liao J., Li C., Yan W., Amani M. (2019). Experimental study of cooling performance of water-based alumina nano fluid in a minichannel heat sink with MEPCM layer embedded in its ceiling. Int. Commun. Heat Mass Transf..

[22] Estelle N.M.P., Estelle P. (2019). Thermal and hydrodynamic performance of a microchannel heat sink with carbon nanotube nanofluids. J. Therm. Anal. Calorim..

[23] Kumar V., Sarkar J. (2019). Effect of different nanoparticle-dispersed nanofluids on hydrothermal-economic performance of minichannel heat sink. J. Therm. Anal. Calorim..

[24] Rajaee F., Amin M., Rad V., Kasaeian A., Mahian O. (2020). Experimental analysis of a photovoltaic/thermoelectric generator using cobalt oxide nanofluid and phase change material heat sink. Energy Convers. Manag..

[25] Alfaryjat A., Miron L., Pop H., Apostol V. (2019). Experimental Investigation of Thermal and Pressure Performance in Computer Cooling Systems Using Different Types of Nanofluids. Nanomaterials.

[26] Neyestani M., Nazari M., Shahmardan M.M., Sharifpur M., Ashouri M., Meyer J.P. (2019). Thermal characteristics of CPU cooling by using a novel porous heat sink and nanofluids. J. Therm. Anal. Calorim..

[27] Sriharan G., Harikrishnan S., Ali H.M. (2021). Experimental investigation on the effectiveness of MHTHS using different metal oxide-based nanofluids. J. Therm. Anal. Calorim..

[28] Arshad A., Jabbal M., Faraji H., Anser M., Talebizadehsardari P., Yan Y. (2021). Thermal process enhancement of HNCPCM filled heat sink: Effect of hybrid nanoparticles ratio and shape. Int. Commun. Heat Mass Transf..

[29] Najafia F., Ramezani D., Sheykh S., Aldaghi A., Taheri A., Sardarabadi M., Passandideh-Fard M. (2021). Fabrication and experimental characterization of a modified heat-sink based on a semi-active/passive cooling strategy with fluid flow and nano-enhanced phase change material. Int. Commun. Heat Mass Transf..

[30] Dammak K., El Hami A. (2021). Thermal reliability-based design optimization using Kriging model of PCM based pin fin heat sink. Int. J. Heat Mass Transf..

[31] Bondareva N.S., Gibanov N.S., Sheremet M.A. (2020). Computational Study of Heat Transfer inside Different PCMs Enhanced by Al_2_O_3_ Nanoparticles in a Copper Heat Sink at High Heat Loads. Nanomaterials.

[32] Zhao L., Xing Y., Liu X. (2020). Experimental investigation on the thermal management performance of heat sink using low melting point alloy as phase change material. Renew. Energy.

[33] Wang Y., Bailey J., Zhu Y., Zhang Y., Boetcher S.K.S., Li Y., Wu C. (2022). Application of carbon nanotube prepared from waste plastic to phase change materials: The potential for battery thermal management. Waste Manag..

